# Regional-Specific Effects of Ovarian Hormone Loss on Synaptic Plasticity in Adult Human *APOE* Targeted Replacement Mice

**DOI:** 10.1371/journal.pone.0094071

**Published:** 2014-04-14

**Authors:** Rebecca C. Klein, Shyla Saini, M-Louise Risher, Shawn K. Acheson, Rebekah L. Fleming, Hannah G. Sexton, H. Scott Swartzwelder, Scott D. Moore

**Affiliations:** 1 Department of Psychiatry and Behavioral Sciences, Duke University Medical Center, Durham, North Carolina, United States of America; 2 Neurobiology Research Lab, Durham Veterans Affairs Medical Center, Durham, North Carolina, United States of America; 3 MIRECC, Durham Veterans Affairs Medical Center, Durham, North Carolina, United States of America; University of Nebraska Medical Center, United States of America

## Abstract

The human apolipoprotein ε4 allele (*APOE4*) has been implicated as one of the strongest genetic risk factors associated with Alzheimer’s disease (AD) and in influencing normal cognitive functioning. Previous studies have demonstrated that mice expressing human apoE4 display deficits in behavioral and neurophysiological outcomes compared to those with apoE3. Ovarian hormones have also been shown to be important in modulating synaptic processes underlying cognitive function, yet little is known about how their effects are influenced by apoE. In the current study, female adult human *APOE* targeted replacement (TR) mice were utilized to examine the effects of human *APOE* genotype and long-term ovarian hormone loss on synaptic plasticity in limbic regions by measuring dendritic spine density and electrophysiological function. No significant genotype differences were observed on any outcomes within intact mice. However, there was a significant main effect of genotype on total spine density in apical dendrites in the hippocampus, with post-hoc *t*-tests revealing a significant reduction in spine density in apoE3 ovariectomized (OVX) mice compared to sham operated mice. There was also a significant main effect of OVX on the magnitude of LTP, with post-hoc *t*-tests revealing a decrease in apoE3 OVX mice relative to sham. In contrast, apoE4 OVX mice showed increased synaptic activity relative to sham. In the lateral amygdala, there was a significant increase in total spine density in apoE4 OVX mice relative to sham. This increase in spine density was consistent with a significant increase in spontaneous excitatory activity in apoE4 OVX mice. These findings suggest that ovarian hormones differentially modulate synaptic integrity in an apoE-dependent manner within brain regions that are susceptible to neurophysiological dysfunction associated with AD.

## Introduction

Alzheimer’s disease (AD) is a cognitive disorder that causes progressive loss of memory and intellectual abilities. It is the most common cause of dementia in individuals over the age of 60. Although both men and women are at risk, sexual dimorphisms in the progression of AD have been shown where women have a greater prevalence and up to a three times greater risk for developing the disease than men [1]–[3].

Genetic risk factors have also been associated with development of late onset and sporadic Alzheimer’s disease. Of the three possible human alleles of the *APOE* gene (ε2, ε3 and ε4), the ε4 allele encodes for a protein that is associated with increased risk of the disease [4], [5]. This cholesterol and lipid transport protein is known to play a critical role in brain development by modulating neuronal growth, maintenance, repair, and protection [6]. In comparison to individuals lacking the ε4 allele, risk of AD increases 3-fold with one allele and 12-fold with two alleles [7]. Conversely, the ε2 allele is associated with a lower risk [8]. Furthermore, studies have also shown a stronger association with the ε4 allele for AD in women [9], [10].

Cognitive decline in AD is often associated with progressive loss of neuronal function, a process that is largely dependent on dendritic spines. Through predominantly excitatory connections, they are responsible for regulating synaptic strength and they are important in maintaining memory throughout the lifespan. Several studies have demonstrated that apoE4 exerts neuropathological effects through changes in dendritic spine morphology. For example, apoE4 transgenic mice and apoE4 AD patients both showed reduced spine density in the dentate gyrus of the hippocampus as compared to the apoE3 genotype [11]. Other studies using male human *APOE* targeted replacement (TR) mice showed reduced spine density in layer II/III cortical neurons at 1, 3, and 12 months of age in apoE4 compared to apoE3 mice, but no differences in the CA1 or dentate gyrus regions of the hippocampus [12]. A more recent study showed a reduction in spine density in CA1 hippocampus and entorhinal cortex from aged (19–21 months), but not adult (7–8 months), female apoE4 TR mice [13]. Taken together, these studies indicate that sex, brain region and the cellular source of apoE are important factors regarding the apoE isoform specific effects on spine morphology.

Structural and functional synaptic plasticity is also highly regulated by ovarian hormones. For example, dendritic spine density is increased by estradiol treatment in cultured hippocampal neurons [14], [15]. It has also been demonstrated that spine density fluctuates across the estrous cycle [16] and ovariectomy induces a decrease in spine density in adult female rats [17]. Electrophysiological studies also show a similar effect of estrous cycle on the magnitude of LTP [18], [19]. Similarly, other authors have shown that the effects of estrogen on NMDA glutamate receptors have been related to the formation of new spines, which could explain the tendency to see an increased number of spines during proestrus, when estrogen levels are highest, in rodents [20].

The beneficial effects of estradiol have also been shown to be dependent upon apoE. For example, estradiol enhanced neuronal outgrowth and synaptic sprouting in wild-type mice, but not in the presence of apoE4 of in apoE knock-out mice [21]–[23]. Other studies have shown regionally-specific changes in apoE expression by estradiol treatment that is also dependent on estrogen receptor subtypes [24], [25]. This complex interaction between apoE and estrogen is a likely contributor to the confounding effects of hormone replacement therapy where some studies have shown beneficial effects [26], [27] while others have not [28], [29].

The amygdala is also impacted by AD, and atrophy in this region is comparable to hippocampal atrophy in early stages of the disease [30]. Furthermore, emotional event memory has been more highly correlated to amygdalar damage than hippocampal volume [31]. Therefore, anxiety and emotional symptoms associated with AD are likely the result of changes in this brain region. Despite this implication, relatively few studies have investigated the role of apoE in synaptic changes in the amygdala. One study demonstrated that apoE4 TR mice have decreased synaptic integrity in the amygdala at 6 months of age [32]; these deficits manifest as early as one month of age, and the presence of apoE2 attenuates the effects of apoE4 [33]. In patients with Alzheimer’s disease, individuals homozygous for the ε4 allele showed greater atrophy in the amygdala compared to patients either lacking the ε4 allele or those with one ε4 allele [34], [35]. It is currently unknown how apoE influences spine morphology and physiology within this region in females.

The aim of the current study was to utilize a relevant mouse model to assess the effects of human *APOE* genotype and loss of circulating ovarian hormones on spine density and electrophysiological activity in the hippocampus and amygdala to test the hypothesis that ovarian hormones are protective against apoE4-associated deficits. We predicted that the presence of estrogen in cycling females would be protective against any genotype differences within intact mice but that ovariectomy would adversely affect synaptic integrity in both genotypes with deficits being significantly more severe in apoE4 mice than apoE3 mice. Given the reported complexities associated with hormone replacement therapy, particularly on the basis of *APOE* genotype, understanding how apoE and the hormonal milieu interact to influence synaptic integrity in females is critical to developing effective therapeutic strategies to treat cognitive dysfunction in genetically susceptible women.

## Materials and Methods

### Ethics Statement

All animal procedures performed in this study were approved by the Durham VA Medical Center Animal Care and Use Committee (protocol number ACUC 1631-001).

### Animals

The *APOE* TR mice used in this study were generated by gene targeting as described previously [13], [36]. Briefly, the TR mice contain human *APOE* genomic fragments in the mouse *Apoe* gene locus via homologous recombination and are therefore under endogenous regulation in a C57B16/J background. Both lines of APOE TR mice contain chimeric genes consisting of mouse 5′-regulatory sequences continuous with mouse exon 1, followed by human exons (and introns) 2–4. Thus, the TR mice regulate apoE expression in the same fashion. Mice used in this study were generated from separate breed pairs (that have been back-crossed to C57B16/J mice 8 times and are therefore >99.6% C57B16/J) for each genotype. All mice were maintained on a standard laboratory rodent diet (LabDiet, St. Louis, MO).

### Surgery

Sham or ovariectomy (OVX) surgery was performed at 5 months of age according to IACUC-approved Durham VA Medical Center Animal Use Protocol. Briefly, mice were continuously anesthetized using isoflurane gas and a subcutaneous injection of buprenorphine (0.1 mg/kg) and carprofen (4 mg/kg) was administered as an analgesic and anti-inflammatory. The lower back was shaved and sterilized and a single, small (<2 cm) incision was made to expose the back muscles. A second incision was made in the muscles above each of the ovaries. Each ovary was pulled out through the incision and cut just above the uterus and then the uterus was replaced. The muscle layer was closed using absorbable suture. Local anesthesia was applied at the incision and the skin was closed using sutures or surgical glue. Mice were placed on a heating pad and allowed to recover and then returned to their original cage and group-housed for the remainder of the study.

Estrous cycle in females was monitored beginning one week after surgery. Vaginal secretions were collected daily for 3 weeks (4–5 days/cycle) in both intact and OVX female mice and examined under a light microscope using a 10x objective. Vaginal secretions in OVX mice had very few cells, confirming the cessation of the estrous cycle.

### Electrophysiology

Acute coronal slices (400 μm thick) were prepared from mice 4–5 weeks post-surgery. Briefly, mice were anaesthetized with isoflurane and decapitated. Brains were quickly removed and placed in ice-cold oxygenated artificial cerebral spinal fluid (ACSF) containing (in mM): 116.4 NaCl, 5.4 KCl, 1.6 MgSO4, 3.2 CaCl_2_, 1 NaH_2_PO_4_, 26.2 NaHCO_3_ and 10 glucose. The brain was glued to the stage of a Vibratome 1000 Plus (Vibratome, Campden, England) and immersed in ice-cold oxygenated ACSF. Slices were transferred to a holding chamber and incubated for at least 1 hour prior to electrophysiological analysis.

Whole-cell patch-clamp recordings were performed in lateral amygdala neurons using patch pipettes with resistances of 3–5 MΩ filled with an internal solution. For the recording of spontaneous excitatory post-synaptic currents (sEPSCs) the internal solution contained (in mM): 140 potassium gluconate, 0.5 CaCl_2_, 2 MgATP, 2 MgCl_2_, 5 EGTA, and 10 Hepes (pH 7.2–7.4). For the recording of spontaneous inhibitory post-synaptic currents (sIPSCs) the internal solution contained (in mM): 140 CsCl, 2 MgCl_2_, 0.5 EGTA, 4 MgATP, 0.5 Tris GTP, 10 Hepes and 5 QX-314 (pH 7.2–7.3). Slices were superfused at room temperature (22–25°C) with ACSF containing in mM: 126 NaCl, 2.5 KCl, 1.3 MgCl_2_, 2.5 CaCl_2_, 1 NaH_2_PO_4_, 25 NaHCO_3_ and 10 glucose. Neurons were clamped at –70 mV and recordings were made using an Axopatch 200B amplifier, filtered at 1 kHz, and sampled at 10 kHz using pClamp 10.1 software (Molecular Devices, LLC, Sunnyvale, CA). The sEPSC and sIPSC intervals and amplitudes were measured using Mini Analysis software (Synaptosoft, Inc. Decatur, GA). Firing properties and electrical excitability were measured using whole-cell current-clamp mode by applying hyper- and depolarizing step pulses for 1 s durations ranging from –0.2 to 0.2 nA in 25 pA increments.

Extracellular field recordings were performed in the CA1 area of the hippocampus using modified techniques described previously [37]. Coronal hippocampus sections were maintained at 30°C in the recording chamber and perfused at flow rate of 4 mL/min with the same ACSF solution that was used for preparing brain slices. A glass micropipette recording tip (2 μm, 2–4 MΩ containing 100 nM NaCl) was placed in the CA1 stratum radiatum and field excitatory post-synaptic potentials (fEPSPs) elicited by stimulating the Schaffer collateral fibers with a concentric bipolar stimulating electrode were recorded using an Axopatch 200B amplifier and pClamp software. For all experiments, input/output curves were generated and the subsequent baseline stimulus intensity was set at a level that elicited 40% of maximal fEPSP response. Baseline was measured for 20 minutes and long-term potentiation (LTP) was induced using a theta burst stimulation protocol consisting of 2 trains of 10 bursts at a frequency of 100 Hz with a 30 sec interval followed by a single stimulus (as used in baseline recordings) every minute for a total of 60 minutes.

### Golgi Staining and Analysis

Neurons were impregnated using the Rapid Golgi Stain Kit (FD Neurotechnologies, Baltimore, MD) according to the package protocol. Briefly, mice were anesthetized using isoflurane, decapitated and the brains were quickly removed and immersed in a 1∶1 mixture of solutions A and B. After 2 weeks, the brains were transferred to solution C for 48 hrs then removed and frozen in tissue freezing medium (Electron Microscopy Sciences, Hatfield, PA), cut into 100 μm thick coronal sections using a cryostat, and mounted onto 2% gelatin coated slides (LabScientific, Inc., Livingston, NJ). Sections were stained with a mixture containing solutions D and E, dehydrated in ethanol, cleared in xylene and coverslipped with Permount. In cycling, sham-operated female mice, brains were isolated during the proestrus phase of the estrous cycle. Golgi-impregnated neurons were visualized using a Neurolucida system and software (MBF Bioscience, Williston, VT) and image stacks were generated using a 100X oil immersion lens. Each image stack was extracted using Image J software (available for free download at http://rsb.info.nih.gov/ij/index.html) and subsequently imported into RECONSTRUCT software [38] (available for free download at http://synapse.clm.utexas.edu) for further analysis. Dendritic spine types were analyzed using an unbiased rating system (developed by W.C. Risher, unpublished) by measuring the length and width of each protrusion with visible connections to the dendritic shaft. Spine densities were determined on the secondary branches of pyramidal-like lateral amygdala neurons and on the apical oblique and second and third order basal dendrites of hippocampal CA1 pyramidal neurons. Data were collected from dendritic segments approximately 10 μm in length and average spine densities were calculated using two separate dendrites from at least 4–5 separate image stacks per animal (N = 4 mice/group). Spine types were determined on the basis of the ratio of the width of the spine head to the length of the spine neck and classified as filopodia, thin, mushroom and stubby.

### Statistical Analysis

For comparison of morphological and electrophysiological endpoints a two-way ANOVA was performed using GraphPad Prism software (GraphPad Software, Inc. La Jolla, CA) with genotype and surgery status as independent variables. Because of the nature of the hypotheses, we anticipated an ordinal interaction. That is, while we expected ovariectomy to affect both apoE3 and apoE4 mice, we hypothesized that apoE4 mice would be more affected than apoE3 mice. Given the well-described difficulty in detecting ordinal interactions using ANOVA [39], analyses were followed by tests of simple main effects using a student’s t-test. Statistical significance was established at alpha <0.05.

## Results

Synaptic plasticity was examined in the within the CA1 region of the hippocampus and the lateral amygdala in adult sham-operated and ovariectomized (OVX) apoE3 and apoE4 TR female mice to compare genotype differences and to determine if the long-term loss of circulating sex hormones differentially influence synaptic structure and function on the basis of apoE. Females underwent surgery to remove the ovaries at 5 months of age and brains were processed 4–5 weeks later. In sham-operated females, analysis was performed at the proestrus stage of the estrous cycle.

### Effects of Ovarian Hormone Loss on CA1hippocampal Spine Denisty and LTP in Female apoE TR Mice

**Figure 1:**
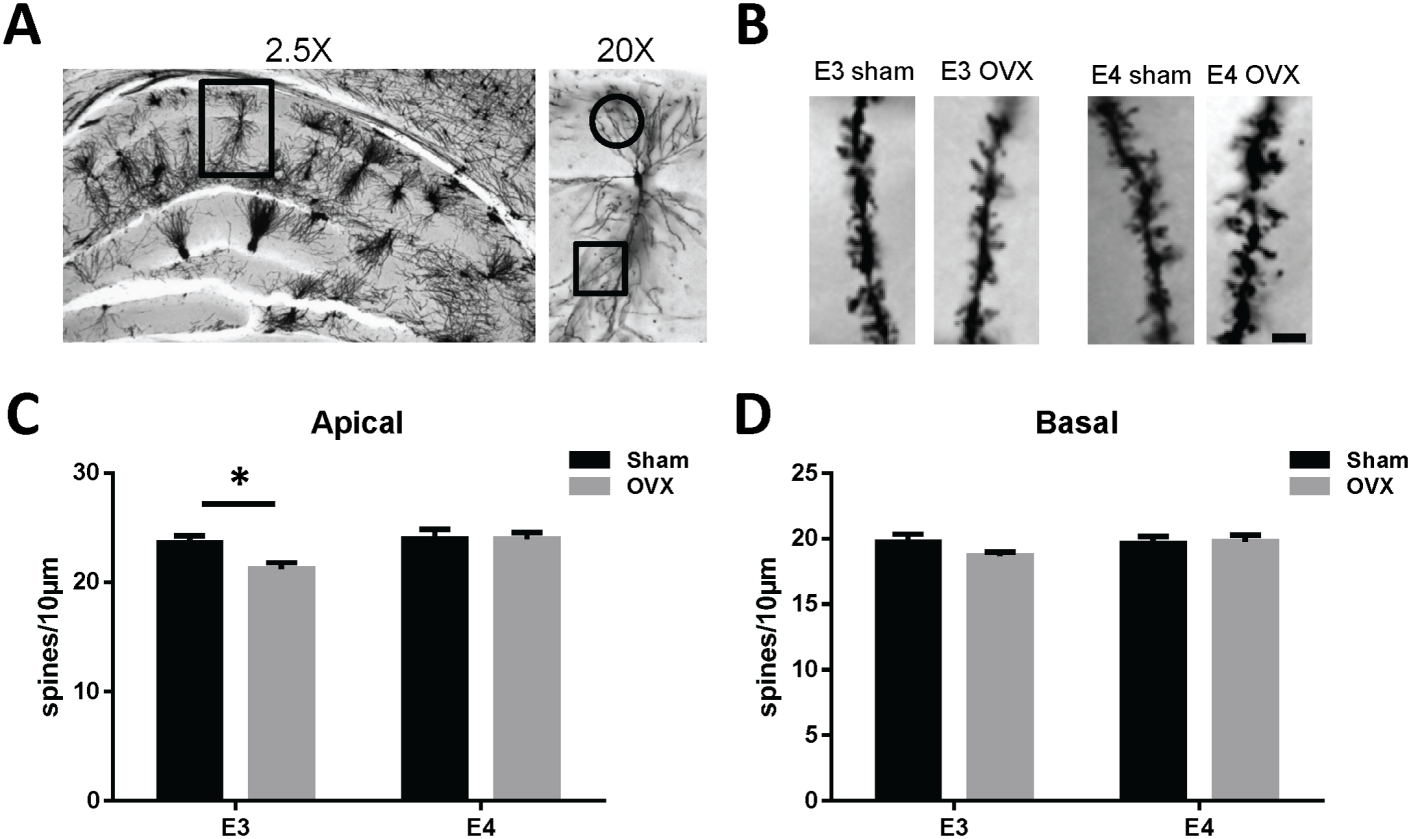
Ovariectomy decreases CA1 apical dendrite spine density in female apoE3 TR mice. (A) Representative photomicrographs of Golgi-stained CA1 pyramidal neurons from a female apoE3 TR mouse brain using a 2.5X and 20X objective. (B) Apical dendritic spines from the designated cohorts using a 100X objective. Dendritic spines were analyzed on apical (square) and basal (circle) dendrites as indicated in the demarcated regions. Scale bar in (B) is 5 μm. Average total spine density on apical (C) and basal (D) dendritic segments within CA1 pyramidal neurons in female apoE TR mice. A two-way ANOVA revealed a significant interaction among the groups for apical spine density (F_1,102_ = 5.678, p<0.019) with post-hoc t-tests showing significantly lower density in apoE3 OVX compared to apoE3 sham (p = 0.0077). There were no significant differences in total basal dendrite spine density. Data represents the mean spine density ± SEM from 6–8 dendritic segments per mouse, with N = 4 mice for each group.

Representative photomicrographs of CA1 pyramidal neurons and plots of the average total spine density from apical and basal dendrites are presented in Figure 1 and values are listed in Table 1. A two-way ANVOA of total spine density resulted in a main effect of genotype among apical dendrites (F_1,102_ = 5.678, p = .019), but no effect of surgery. Simple main effects analysis revealed a significant reduction in OVX E3 mice relative to sham E3 mice (p = 0.0077). Conversely, there were no significant surgery effects on total spine density among basal dendrites. In addition, there was no effect of genotype among sham-operated mice on either apical or basal dendrite spine density. Apical and basal dendrite spine type densities were assessed and are presented in Table 1. There were no significant genotype or surgery effects among these spine types.

**Table 1:** Total and spine-type density from hippocampal (Hpc) CA1 pyramidal neuron apical and basal dendrites and lateral amygdala neurons in sham-operated and OVX apoE TR mice.

Region/Type	E3 Sham	E3 OVX	E4 Sham	E4 OVX
**Hpc apical**				
Total Density	23.6±0.7	21.2±0.5*	24.0±0.9	24.0±0.6
Filopodia	2.0±0.3	1.6±0.3	2.0±0.5	1.7±0.3
Thin	12.0±0.8	10.4±0.8	12.7±0.8	11.6±0.8
Mushroom	8.1±0.7	7.5±0.7	7.0±0.5	8.3±0.7
Stubby	1.5±0.3	1.7±0.2	2.2±0.3	2.4±0.3
**Hpc basal**				
Total Density	19.7±0.6	18.7±0.3	19.6±0.5	19.8±0.5
Filopodia	0.9±0.2	0.9±0.3	1.2±.04	0.8±0.3
Thin	9.1±0.8	9.9±0.9	10.5±0.6	10.3±0.8
Mushroom	6.7±0.6	5.5±0.8	5.1±0.6	6.4±0.6
Stubby	2.5±0.4	2.4±0.4	2.8±0.4	2.4±0.3
**Lateral amygdala**				
Total Density	18.3±0.6	18.2±0.5	18.2±0.5	20.4±0.6*
Filopodia	2.3±0.3	1.8±0.4	1.4±0.2	2.6±0.4*
Thin	10.5±0.7	9.9±0.7	10.0±0.6	12.4±0.6*
Mushroom	2.8±0.4	3.3±0.4	3.6±0.5	2.4±0.4**
Stubby	2.7±0.3	3.2±0.3	3.2±0.3	3.0±0.4

Spine density (spines/10 μm) is presented as mean ± SEM, *p<0.001, **p<0.05 for OVX relative to sham within the same genotype.

**Figure 2:**
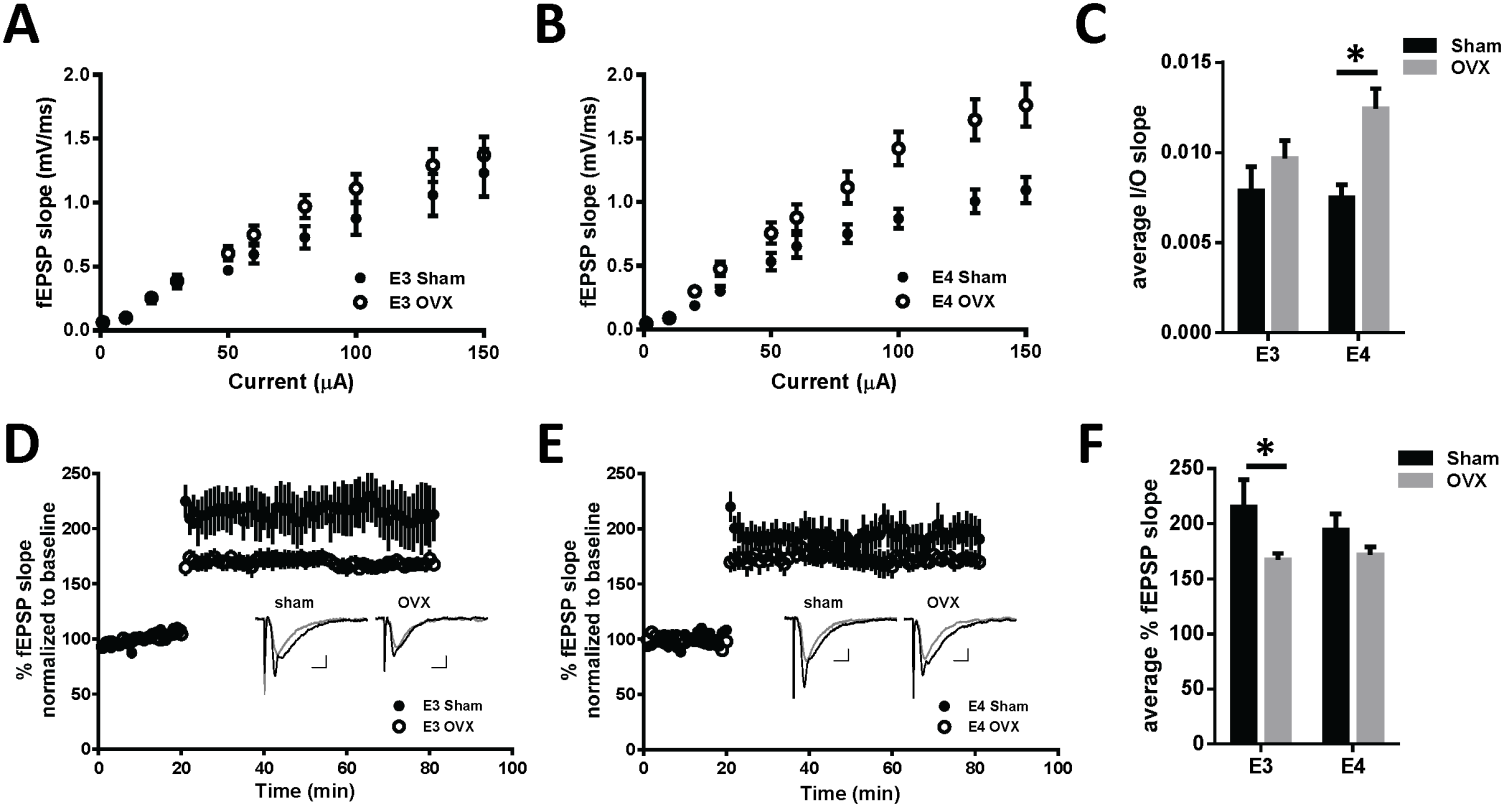
Ovariectomy differentially influences CA1 hippocampal synaptic transmission in female apoE TR mice. Input-output curves presenting fEPSP slope in response to increasing stimulus input in apoE3 (A) and apoE4 (B) sham and OVX mice. (C) Average I/O slopes measured between 1 μA and 150 μA resulted in a significant increase in I/O slope in apoE4 OVX mice relative to sham (p = 0.0033). Long-term potentiation expressed as percent fEPSP slope normalized to baseline in apoE3 (D) and apoE4 (E) sham and OVX mice. Inset: representative traces of fEPSPs 5 min. before (gray line), and 40 min. after (black line) LTP induction from the designated mice. (F) Summarized magnitude of LTP representing the average percent potentiation of the last 20 minutes of recording. A two-way ANOVA resulted in a significant main effect of surgery (F_1,38_ = 8.04, p<0.0073) and post-hoc tests revealed a significant reduction in LTP magnitude in apoE3 OVX mice relative to sham (*p = 0.019). Data are presented as mean ± SEM from 1–2 slices recorded from the following number of mice: apoE3 sham n = 6; apoE3 OVX n = 8; apoE4 sham n = 7; apoE4 OVX n = 7.

Basal synaptic transmission and long-term potentiation were measured in order to assess the effects of genotype and surgery status on functional activity within the CA1 region of the hippocampus. Input-output (I/O) curves were generated by measuring fEPSP slope in response to increasing stimulus intensity and are presented in Figure 2. ApoE3 sham and OVX mice showed comparable responses to increasing stimulus intensity, whereas apoE4 OVX mice demonstrated an increase in fEPSP slope relative to sham. A two-way ANOVA of the average I/O slopes measured between 1 μA and 150 μA (Figure 2C) resulted in a significant main effect of surgery (F_1,39_ = 8.647, p = 0.0055) with simple main effects analysis revealing a significant increase in I/O slope in apoE4 OVX mice relative to sham (p = 0.0033) but not in apoE3 mice (p = 0.317).

Long-term potentiation was successfully induced within all of the groups as shown in Figure 2D and 2E. A two-way ANOVA comparing the average percent potentiation of the last 20 minutes of recording (Figure 2F) resulted in a main effect of surgery (F_1,38_ = 8.038, p = 0.0073). Simple main effects analysis revealed a significant reduction in LTP magnitude in apoE3 OVX mice relative to apoE3 sham (p = 0.019). Although the trend was similar among apoE4 mice (OVX<Sham), it was not significant (p = 0.136). There were also no significant genotype differences between sham-operated mice (p = 0.304).

### Effects of Ovarian Hormone Loss on Lateral Amygdala Spine Denisty and Neuronal Activity in Female apoE TR Mice

**Figure 3:**
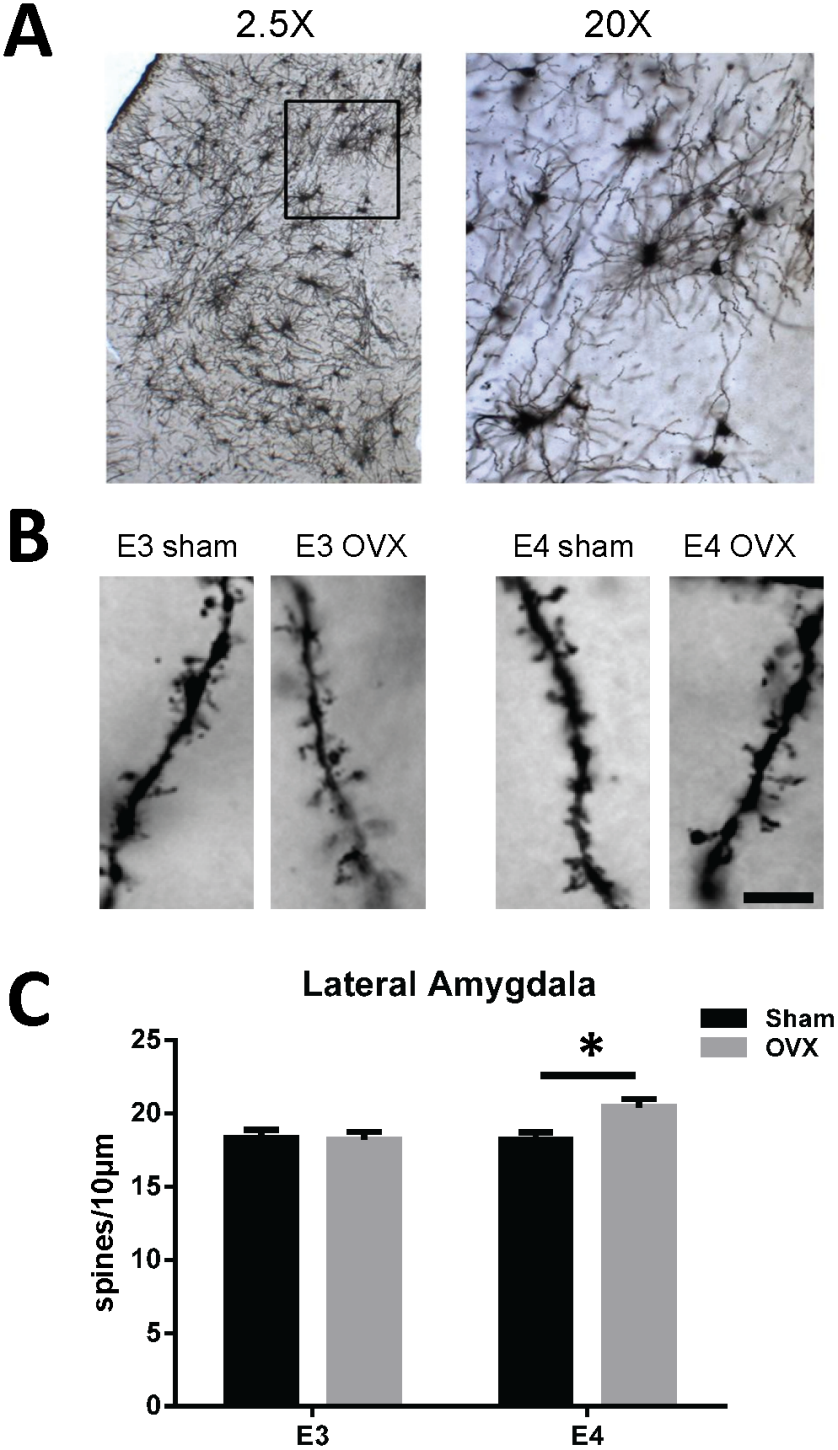
Ovariectomy increases lateral amygdala spine density in female apoE4 TR mice. (A) Representative photomicrographs of Golgi-stained lateral amygdala neurons from an adult female apoE4 TR mouse brain using a 2.5X, 20X and (B) dendritic segments from the designated cohorts using a 100X objective (scale bar is 5 μm). (C) A two-way ANOVA of average total spine density resulted in a significant interaction (F_1,105_ = 4.740, p = .0317) and post-hoc tests revealed a significant increase in spine density in apoE4 OVX mice relative to sham (p = 0.0038). Data represent the mean spine density ± SEM from 8 dendritic segments per mouse, with N = 4 mice for each group, except for apoE3 OVX mice, N = 3.

Representative photomicrographs depicting pyramidal-like neurons and dendritic spines in the lateral amygdala are illustrated in Figure 3A and 3B. Plots of the average total spine density are presented in Figure 3C and the values are listed in Table 1. A two-way ANOVA resulted in a significant interaction on total spine density (F_1,105_ = 4.740, p = .0317). Simple main effects analysis revealed a significantly higher total spine density in apoE4 OVX mice relative to sham (p = 0.0038). Similar to the hippocampus, there were no significant genotype differences within sham-operated mice. Spine type densities were also measured and the values are preseneted in Table 1. There was a significant interaction on measures of filopodia (F_1,105_ = 6.601, p = 0.012), thin (F_1,105_ = 4.942, p = 0.028) and mushroom (F_1,105_ = 4.653, p = 0.033) spine density. Simple main effects analysis revealed a significant increase in filopdia (p = 0.0064) and thin (p = 0.0065) spine density and a decrease in mushroom spine density (p = 0.028) in apoE4 OVX mice compared to apoE4 shams. There were no signficant differences in total or spine type densities between apoE3 OVX and sham-operated mice.

**Figure 4:**
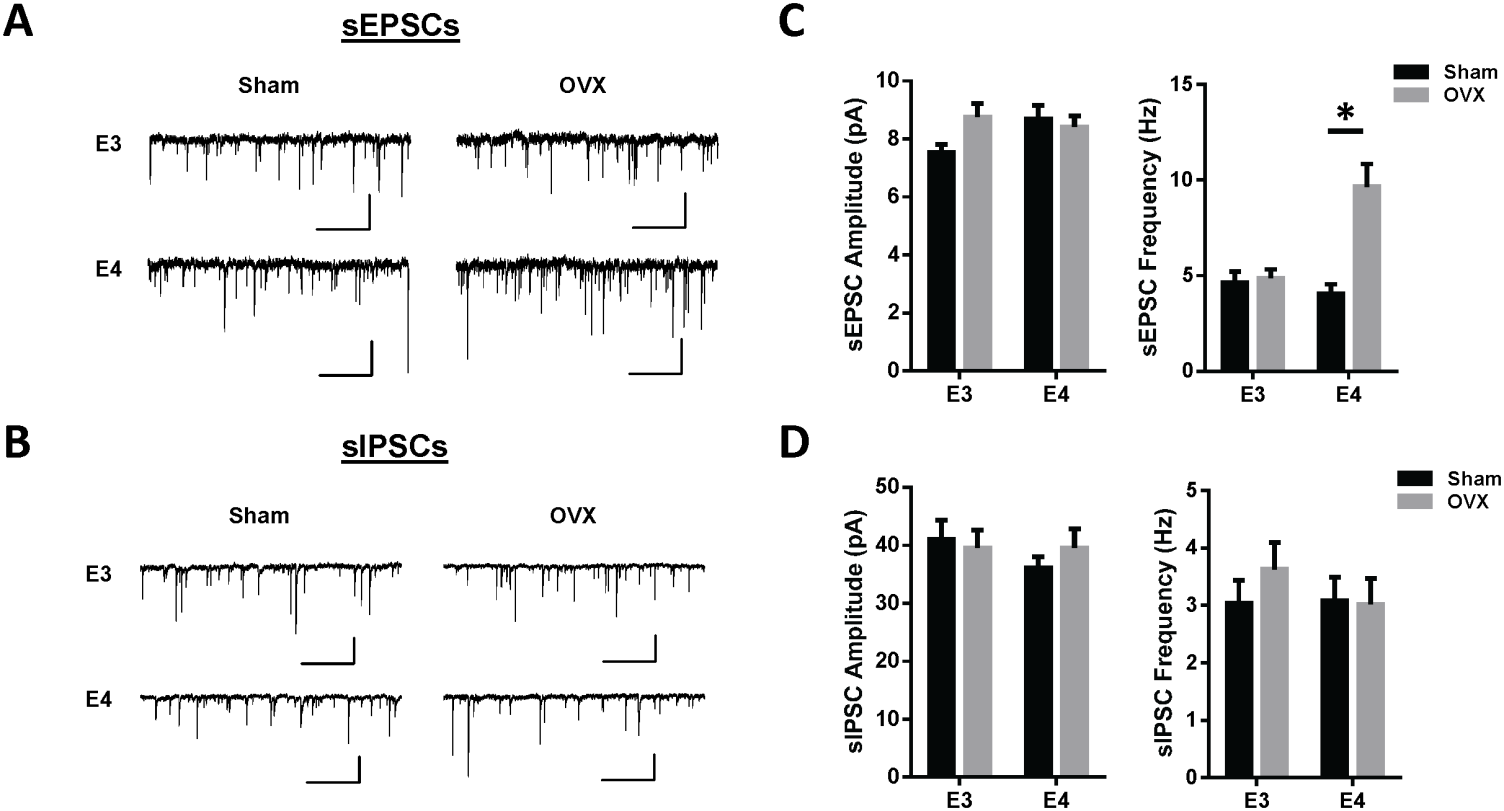
Ovariectomy increases sEPSC frequency in female apoE4 TR mice. Representative traces and average sEPSC (A) and sIPSCs (B) amplitudes and frequencies. (C–D) A two-way ANOVA of sEPSC frequencies resulted in a significant interaction (F_1,62_ = 10.41, p = 0.002). Post hoc t-test analysis revealed apoE4 OVX mice had a significantly higher sEPSC frequency than apoE4 sham mice (p = 0.0004) while there was no effect of OVX in apoE3 females. Values represent mean ± SEM, n = 12–20 neurons from 6–8 mice per group. Scale bar is 10 pA, 1 sec in (A) and 50 pA and 1 sec in (B).

Whole cell patch-clamp recordings were obtained from pyramidal neurons within the lateral amygdala in order to assess the effects of genotype and surgery status on functional activity within this region. Spontaneous excitatory post-synaptic current (sEPSC) and inhibitory post-synaptic current (sIPSC) amplitudes and frequencies were measured from 2-minute continuous recordings from 2–4 neurons from 6–8 mice per group. Representative traces and comparison of genotype and surgery status on the mean sEPSC and sIPSC amplitudes and frequencies are presented in Figure 4A and 4B, respectively. A two-way ANOVA of sEPSC frequencies resulted in a significant interaction (F_1,62_ = 10.41, p = 0.002). Simple main effects analysis revealed a significantly higher sEPSC frequency in apoE4 OVX mice relative to sham apoE4 mice (p = 0.0004). There were no significant genotype differences within sham-operated mice and there was no significant effect of OVX in apoE3 females. There were no significant differences between any of the groups on sEPSC amplitude. In addition, there were no significant differences between any of the groups on sIPSC amplitude or frequency.

## Discussion

The purpose of the present study was to examine the interaction between *APOE* genotype and the long-term loss of circulating ovarian hormones on spine density and functional activity in the hippocampus and amygdala from female human *APOE* TR mice. The data presented herein revealed significant regionally specific genotype-dependent effects in ovariectomized mice, with apoE4 mice displaying an overall increase in excitatory neurotransmission. In the hippocampus, OVX resulted in a significant increase in the level of basal synaptic transmission in apoE4 mice. OVX also had a genotype-dependent effect in the lateral amygdala, resulting in increased spine density and spontaneous excitatory activity only in apoE4 OVX mice relative to sham. This is the first study that has demonstrated that there is a significant change in synaptic transmission in response to hormone loss in the human *APOE* TR mice. These findings may help explain why human *APOE4* females with AD fail to benefit from hormone replacement therapy [28], [29].

As expected, there were no significant genotype differences within sham-operated mice on any of the outcome measures. These findings are consistent with previous studies showing 1) a lack of genotype effect on CA1 hippocampal spine density in adult (7–8 months) female apoE TR mice [13], 2) a lack of differences between apoE3 and apoE4 on LTP in the dentate gyrus among aged (18–24 months) female mice [40] and 3) a lack of differences between apoE3 and apoE4 on NMDA-independent LTP in the CA1 region in male mice [41].

We initially expected that apoE4 mice would have a more severe decline in synaptic integrity compared to apoE3 mice in response to OVX due to the protective actions of estrogen. However, based on our results, it appears that apoE3 and apoE4 mice respond in opposing ways to ovarian hormone loss. It may be that apoE3 mice are more susceptible to the effects of estrogen loss and that apoE4 mice are more susceptible to the effects of progesterone loss or the combined effects of both hormones. Both estrogen and progesterone regulate neuronal morphology and function via opposing mechanisms where the effect of estrogen has largely been associated with an increase in neural excitability while the effects of progesterone regulate inhibitory tone. In the hippocampus, estrogen increases CA1 spine density [42], [43] and long-term potentiation [44]. In contrast, estrogen has been shown to reduce excitability in the amygdala by decreasing excitatory post-synaptic potentials in the basolateral amygdala [45] and by reducing excitatory afferent connections to the medial amygdala [46]. Therefore, apoE3 mice may be sensitive to estrogen loss which leads to a decrease in spine density and thus a reduction in hippocampal LTP while apoE4 mice may be more sensitive to the combined loss of estrogen and progesterone, thus leading to and increase in spine density and greater excitability in the amygdala. Due to the lack of increase in spine density in the hippocampus, future studies are warranted to explore whether the apoE4-associated increase in hippocampal excitability is due to an increase in post-synaptic AMPA receptor expression or an increase in presynaptic inputs.

It is also possible that the loss of estrogen and/or progesterone in apoE4 mice leads to hyperexcitablity via an enhanced response to stressful stimuli. Synaptic plasticity in the amygdala has been associated with increased susceptibility to stress [47]. For example, immobilization stress leads to an increase in spine density in the basolateral amygdala [48], [49]. Our observed increase in total spine density in apoE4 OVX mice is consistent with this effect. Further, the significant changes in filopodia and thin spine type densities are consistent with the formation of new dendritic spines as well as the types of spines present when estrogen levels are low [20].

Interestingly, the hyperexcitability observed in apoE4 OVX mice is consistent with a recent report showing increased seizure phenotype in aged female apoE4 TR mice [50]. In addition, rodent models have shown that excess hippocampal activation contributes to age-related memory impairments [51], [52] and that treatment with low doses of antiepileptic drugs improved memory performance [53], [54]. Behavioral deficits due to impairments in hilar GABAergic interneuron function have also been shown in apoE4 females as a function of age [55]. While our results did not show any effect of OVX on inhibitory activity in the amygdala, it remains to be determined how GABAergic tone in the hippocampus is modulated. Future studies involving the reintroduction of each hormone and combinations thereof are necessary to further understand the complex interaction between apoE and ovarian hormones on neuronal excitability and inhibition.

Another potential explanation for the differential apoE3 and apoE4 finding may be due to *de novo* estrogen synthesis. Previous studies have shown positive correlations between circulating estradiol and spine density in CA1 apical dendrites [56], [57]. Recent evidence suggests, however, that estrogen is synthesized in select brain regions [58] even following OVX [59]. Studies have shown the presence of aromatase, the final enzyme in the estrogen synthesis pathway, in a population of CA1 pyramidal cells of OVX female rhesus monkeys both with and without long-term cyclic estradiol treatment [60]. Furthermore, inhibition of aromatase in the brain decreases spine density in the hippocampus, suggesting that it may be neural estradiol that affects synaptic integrity [56]. This could explain the unexpected result, if OVX did not in fact have the expected effect on brain estrogen levels in apoE4 mice. Future studies are warranted to determine if there are genotype differences associated with aromatase activity and brain estradiol levels in the *APOE* TR mice.

A third explanation for the differential result may be due to the differences in estrogen receptor (ER) subtype localization. Estradiol action is mediated by two distinct receptors: ERα and ERβ. While both receptor subtypes are present in several regions of the brain, ERα may be the predominant type in the hippocampus whereas ERβ is localized to nuclei within the amygdala [61]. Since apoE4 has been hypothesized to have a dominant negative effect on neurodegeneration [62], differences in the interaction between estrogen receptors and apoE expression may account for differences seen in the two regions. For example, it has been demonstrated that activation of ERα increases apoE protein expression, whereas activation of ERβ decreases apoE expression [25]. Thus, the inhibitory actions of estrogen on apoE expression via ERβ activation in the amygdala, and the loss of apoE expression via activation of ERα in the hippocampus may be beneficial against the negative effects on synaptic integrity associated with apoE4. This hypothesis, too, would be useful to evaluate in future studies by quantifying regional apoE levels in response to the hormonal environment.

Finally, while the present study focused on the interaction between apoE and estrogen in a female mouse model, the effects of androgens and androgen receptors may also play a role in the genotype specific outcomes. For example, it has been shown that testosterone treatment is protective against apoE4-associated deficits on a spatial learning and memory task in females and this effect may be partially mediated by conversion to 17β-estradiol since dihydrotestosterone was less efficacious than testosterone [63]. Androgens mediate their effects by binding to ARs [64], [65] and it has been hypothesized that apoE4 may disrupt androgen and AR-mediated pathways due to a stronger interaction between apoE4 with AR, leading to a loss of AR signaling/function [66]. In this case, the increase in synaptic plasticity we observe in the OVX apoE4 mice may be due to a decrease in apoE levels following OVX, leading to a decrease in apoE4-AR interaction and thus an increase in AR signaling. This would account for the genotype differences we observe, where the effects depend on not only estrogen, but testosterone as well.

In conclusion, results from this study suggest that the presence of circulating ovarian hormones plays a significant role in modulating synaptic transmission on the basis of *APOE* genotype. Importantly, the long-term loss of ovarian hormones varies significantly depending on genotype and brain region, where the CA1 region of the hippocampus is affected in apoE3 mice and the lateral amygdala in apoE4 mice. Future studies aimed at examining the effects of reintroduction of estrogens and/or progesterone are warranted in order to identify the specific hormones and time-course involved in these outcomes. Further understanding how ovarian hormones differentially impact the neurocircuitry of memory and emotion by *APOE* genotype is critical toward developing effective treatments for age-related neuronal dysfunction among postmenopausal women.
